# Proposal of a magnetic resonance technique for the evaluation of the calcaneofibular ligament minimizing false positive results

**DOI:** 10.1186/1475-925X-13-168

**Published:** 2014-12-16

**Authors:** Ibevan A Nogueira, Annie F Frère, Alessandro P Silva, Terigi A Scardovelli, Silvia RMS Boschi, Heverton C Oliveira

**Affiliations:** Núcleo de Pesquisas Tecnológicas, Universidade de Mogi das Cruzes, Mogi das Cruzes, São Paulo, Brazil; Departamento de Radiologia, Universidade Federal de São Paulo, São Paulo, São Paulo Brazil

## Abstract

**Background:**

Magnetic resonance (MR) techniques used to detect lesions of the ligament complex for articulation of the ankle lack the desired accuracy for the study of the calcaneofibular ligament (CFL). The lack of sensitivity of the conventional techniques is due to variations in the dimensions of the CFL. The best results are obtained when the image plane is oriented parallel to the ligament. This study aims to develop a model that addresses the width, length and angle parameters of the CFL and the orientation of the MR image plane, and thus determine a technique in the oblique transversal plane with the foot in anatomical flexion, that is adequate for the majority of patients.

**Method:**

To determine this orientation and adapt it to the majority of people, images of the articulation of the ankle in the 3D isotropic, volumetric, sagittal plane of 100 volunteers were taken using the MR technique. None of the volunteers had a clinical history of ligament lesions, serious pathologies, or surgeries. A measurement of the length, width, and angle of the CFL relative to the sole of the foot was performed using the MR tools. A virtual model was developed that simulated the visualization of the CFL in the oblique transversal image plane from 35° to 45° using the CFL dimensions of 100 volunteers. The comparison of the simulations with the reconstructed images validated the model and permitted the calculation of the agreement and sensitivity of each technique in the detection of the complete CFL.

**Results:**

Using the simulator, it was possible to obtain the limit angle for complete CFL visualization as a function of its dimensions for any angle of the oblique transversal image plane of the MR.

**Conclusion:**

The results suggest that a single image acquisition technique in the oblique transversal plane at 38° with the foot in anatomical flexion would serve the majority of patients.

## Background

The ligament complex of the ankle is composed of anatomical structures of various ligaments that are difficult to analyze. Among these structures, the calcaneofibular ligament (CFL) is frequently injured in young adults, especially individuals involved in sports such as basketball and football. This ligament is also the most vulnerable during inversion of the ankle [1]. According to Park et al. [2], 85% of ankle sprains involve the lateral complex of the ankle.

Magnetic Resonance (MR), a non-invasive and well accepted imaging technique [3], has been used for the evaluation of ligament structures due to its capacity to provide excellent contrast between the soft tissues (ligament and muscle). However, current techniques lack the desired accuracy for the study of the calcaneofibular ligament (CFL). Certain studies [4, 5] have shown that the use of an improper image plane leads to an imprecise diagnosis. Therefore, various researchers [2, 6–8] have studied the efficiency of MR techniques that use coronal, axial, and sagittal image planes for better visualization of these images. Muhle et al. [9] and Boonthathip et al. [10] obtained promising results with MR in the oblique transversal plane; however, they also addressed the need for more studies.

The lack of sensitivity of these techniques can be explained by variations in the dimensions of the CFL. In fact, Mayerhofer et al. [11] proved that the best results are obtained when the plane of the MR image is parallel to the ligament. However, as shown by Golanó et al. [12], the angle of the CFL with the sole of the foot has a different value for each person. It was already proven [4, 5] that the orientation of this angle does not always coincide with the standard orientation of the image plane and therefore influences its visualization. However, a technique that provides good results for the majority of patients has not been determined. In addition, it was not investigated whether variations in the other dimensions of this ligament are relevant in the detection, although Boonthathip et al. [10] detected relevant variations in the dimensions of the length and width of the CFL of volunteers investigated with the foot in the same position. However, it is difficult and slow to use experimental procedures when many independent variables are involved. Mathematical and computer models are useful in such investigations, as shown Nogueira et al. [13], who successfully developed a model to study the kinematics of the articulation of the knee and by Chuang et al. [14] who developed a model of the brain to investigate the individualized calibration for NIRS measurements.

Thus, in this investigation, to consider the influence of the width, length, and angle of the CFL, a model that addresses these parameters and the orientation of the MR image plane was developed. A technique was thereby determined, using the oblique transversal plane with the foot in an anatomical flexion that evidenced the whole CFL for the majority of healthy subjects.

## Method

### Research volunteers

Male and female adult volunteers with ages between 21 and 50 years were selected by the doctors of the Department of Radiology of the Lumen Diagnostics Centre.

The inclusion criteria were volunteers without a history of ankle sprains and without CFL lesions, without fractures, congenital diseases, or orthopedic surgeries involving the ankle and foot. Cases where fracture of the CFL was detected in the volumetric acquisition of the images were excluded from the sample. The acquisition of volumetric isotropic images of the volunteers in the sagittal plane was performed. The images of these volunteers were reported and sent to the researchers (CAE 03556912.0.0000.5497).

### Procedures

The volumetric isotropic MR images of the 100 volunteers were first used to determine the dimensions of the CFL using the tools of the supervisory system of the Achieva Magnetic Resonance device. The measurements were performed 3 times by two technicians specializing in the musculoskeletal system.

Next, a computational model was developed that represents the CFL relative to the image plane and determines whether there is complete visualization of this ligament. The model can consider all of the dimensions of the ligament with the foot in the anatomical position and simulates the visualization of any orientation of the oblique transversal image plane.

To validate the computerized model, the CFL and its visualization were initially simulated considering the oblique transversal image plane at 35°. This technique was chosen because 35° is the CFL angle with the highest prevalence in the studied population. The CFL morphometric data from the volunteers were used for the simulation.

As follow, the images were acquired in oblique plane at 35° and the isotropies images from the 100 volunteers were reconstructed in the same image plane. The accuracy of image detection was obtained directly in the oblique plane at 35° and compared with the reconstructed images accuracy. This comparison was also accomplished with the simulated images using the C contingency test.

After validating the model, simulations were performed considering the oblique image plane at 36°, 37°, 38°, 39°, 40°, 41°, 42°, and 45°. The accuracy of detection was calculated to determine the best technique.

To test the method, reconstructions of the 100 volumetric isotropic images were performed in the oblique transversal plane at 38°, which was considered to be the most efficient by the simulator, and at the 45° angle used by many clinics. These images, without technical information, were evaluated at work stations by 2 Radiology Technicians specialized in Magnetic Resonance of the musculoskeletal system. The examiners analyzed the images and, in consensus, evaluated which images visualized the entire length of the CFL.

An analysis of agreement of the accuracy obtained across the various techniques was performed, both of the reconstructed images and of the simulations using the C contingency test. The reports from the doctors of the Lumen Diagnostic Centre were considered to be the gold standard.

#### Image bank

**Figure 1:**
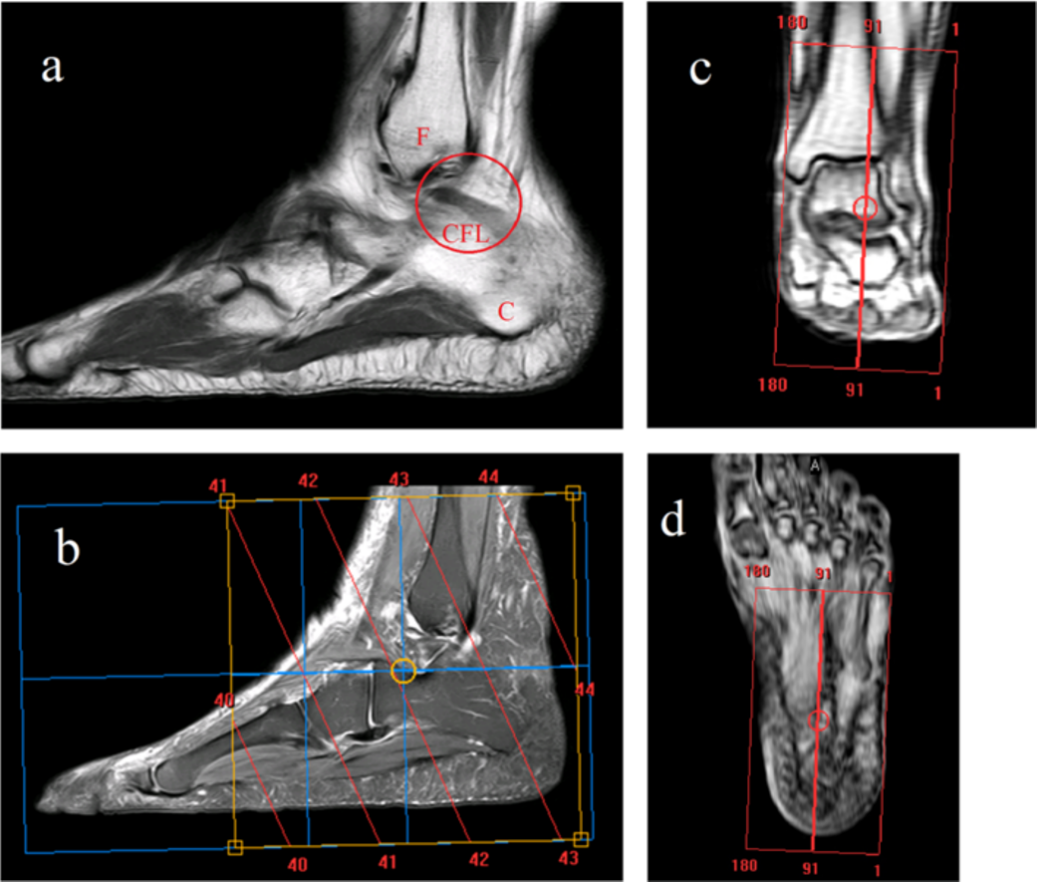
**Volumetric planning to image acquisition.** Figure 1 **a** highlights the CFL anatomic region (F = Fibular bone and C = Calcaneus bone); Figure 1 **b-d** shows the 3D sagittal, coronal and transversal volumetric planning respectively.

The images were acquired using a 3D volumetric isotropic proton density (PD) weighted turbo spin echo (PDW-TSE) sequence without fat saturation in the sagittal plane (TR/TE, 1000/30 ms; turbo factor 20; field of view 150 mm; thickness of voxel cut size (0.5)^3^; matrix 300 × 250; number of excitations 2; and acquisition time 3 minutes and 10 seconds) (Figure 1a, 1b, 1c, 1d). An Achieva Magnetic Resonance device (Philips Medical System; Cleveland, OH, USA) was used with a “Sense Ankle Foot” bobbin with 8 (eight) channels for the transmission and reception of signals.

**Figure 2:**
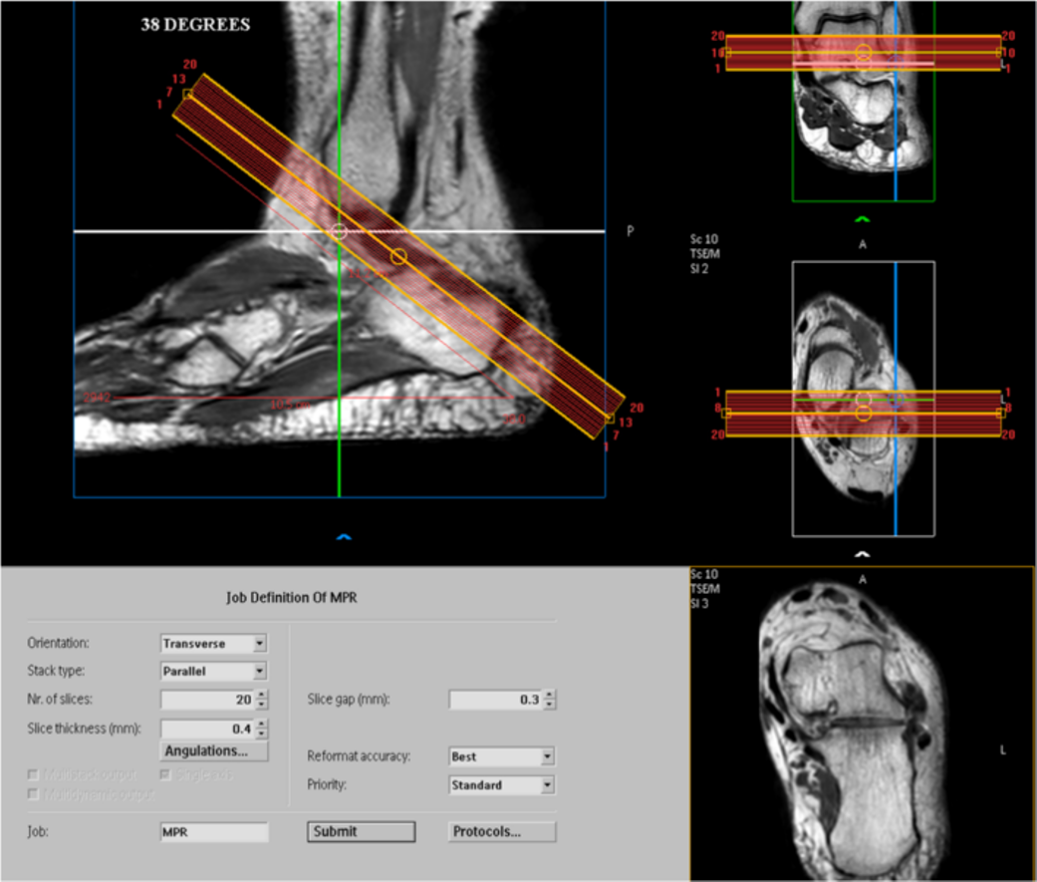
**Supervisory system of the MR device.** Supervisory system tools of the Achieva device for the reconstructed image (MPR) in the oblique transversal plane at 38°.

The volunteers were positioned in the supine position with the arms extended along the body, and the sequences were performed with free breathing. During the exams, the position of the foot relative to the ankle was maintained at an angle of 90°, using immobilizers and special foams to maintain an anatomical position.The 3D sagittal volumetric images of the 100 volunteers permitted multiplanar reconstructions. For these reconstructions, the multiplanar reconstruction (MPR) commands were used, followed by the orientation of the oblique transversal plane and then the line setting function to select an angle of 35°, 38° and 45° (Figure 2).

#### Ethical approval

Before to start this research with the specific, this research project was approved by the Ethics Committee advice in Research involving humans at the University of Mogi das Cruzes (CAAE-0151.0.237.000-10, process CEP/UMC-157/2010).

All participants were informed about the aims, and methodology of the study, as well as about the privacy of research subjects and confidentiality of their personal information, and institutional affiliations of the researcher.

#### Determination of the CFL measurements

**Figure 3:**
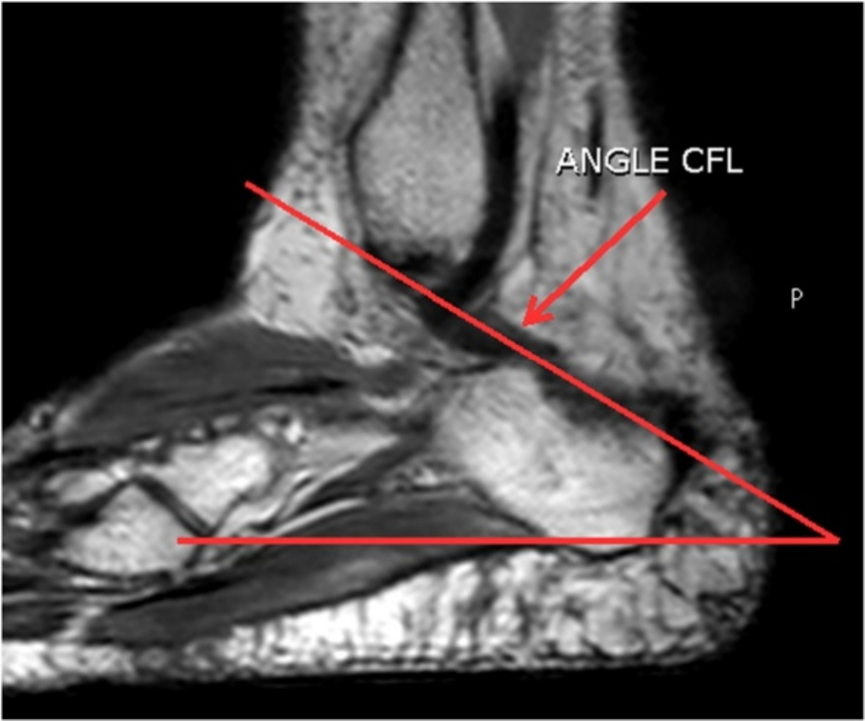
**Measure the angle of the CFL 3D sagittal plane.** Trace the line in the sagittal plane, parallel to the horizontal flat surface, and another line was traced parallel to the plane of the ligament investigated. The angle between these lines was calculated using the tools of the MR device.

**Figure 4:**
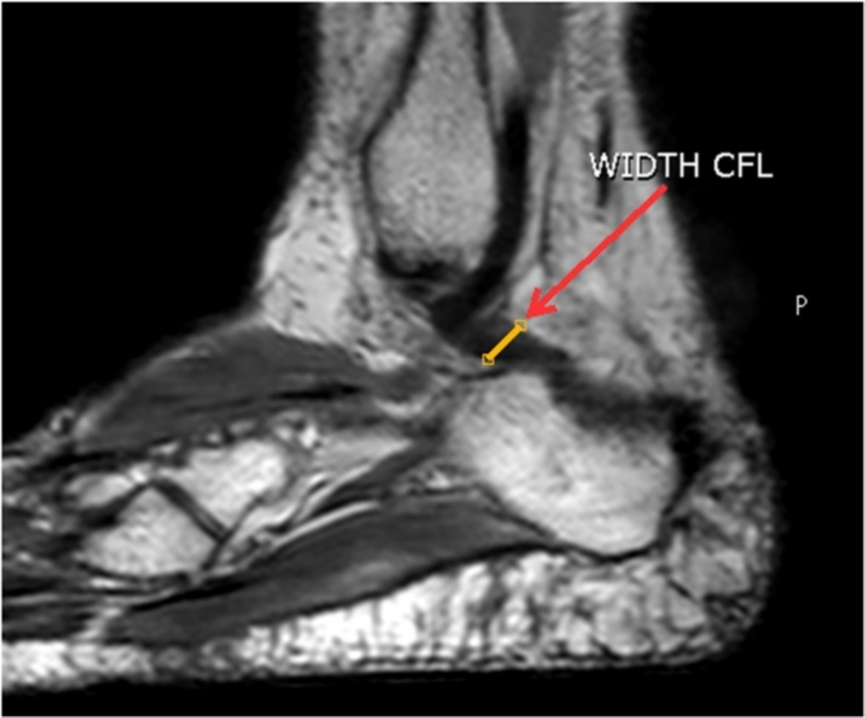
**Measuring the width of the CFL.** Traced line in the widest region of the ligament obeying the anatomical plane to calculate the CFL width.

**Figure 5:**
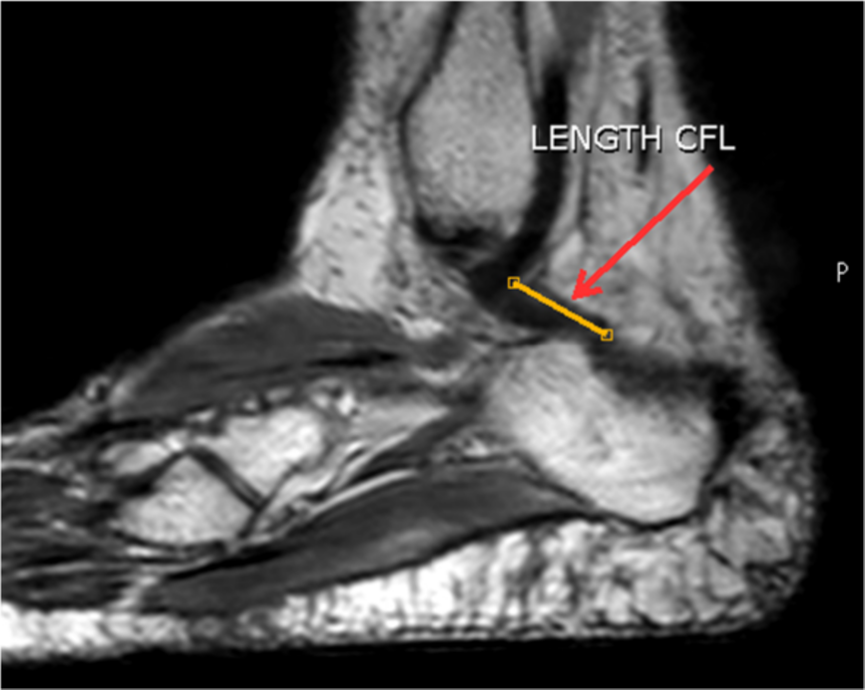
**Measuring the length of the CFL.** Traced line between the distal point of origin of the fibula and the point of insertion of the ligament on the calcaneus to calculate the CFL length.

Images were used in the volumetric sagittal plane to measure the average angle, length, and width of the CFL of each volunteer.To measure the angle, using the “Line Setting” tool with angle option, a straight line was traced in each image in the sagittal plane, parallel to the horizontal flat surface, and another line was traced parallel to the plane of the ligament investigated (Figure 3). After this command, the angle between these lines was calculated using the tools of the MR device.To measure the width of the CFL, a line was traced in the widest region of the ligament obeying the anatomical plane. The MR system tools permitted tracing and calculating the CFL width in mm (Figure 4).To measure the length of the CFL, the distal point of origin of the fibula and the point of insertion of the ligament on the calcaneus were marked. The value of the length between these two points was calculated using the MR device system tools (Figure 5).

#### Model for the simulation of the positioning of the CFL

**Figure 6:**
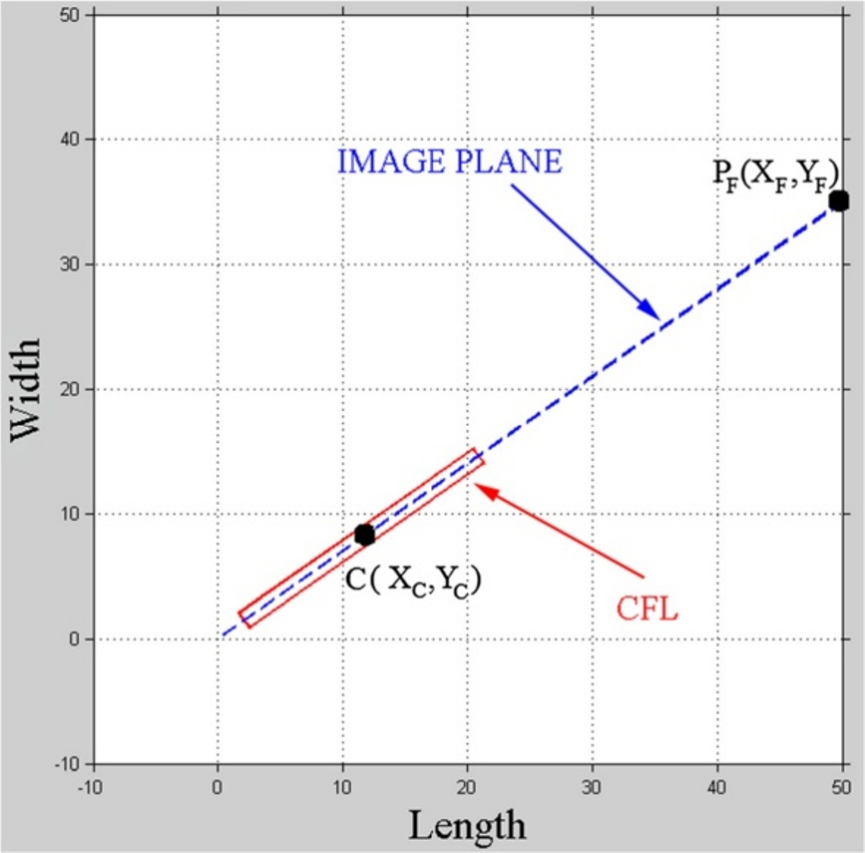
**Model for visualization simulation of CFL.** The CFL was represent by red rectangle and the image plane by blue dotted line, with coordinates of the center of the CFL (C) and the end point of the image plane (IP), moreover the angle of the CFL and the image plane are coincident at 35°.

The programming and graphic development module of MatLab® was used to simulate the limits of complete visualization of the calcaneofibular ligament (CFL) as a function of the angle of the image plane.The variable dimensions (length, width, and angle) are input into the model by the user. The application considers the CFL as a rectangle represented by continuous red lines. Initially, the central axis of the rectangle is aligned with the image plane, represented by a dotted blue line (Figure 6). The rectangle is rotated until its upper vertex coincides with the image plane, thus determining the maximum angular variation (β) that permits complete visualization of the CFL. The application indicates whether there was complete visualization for the CFL angle and the image plane angle, considering the width and the length of the ligament.

**Figure 7:**
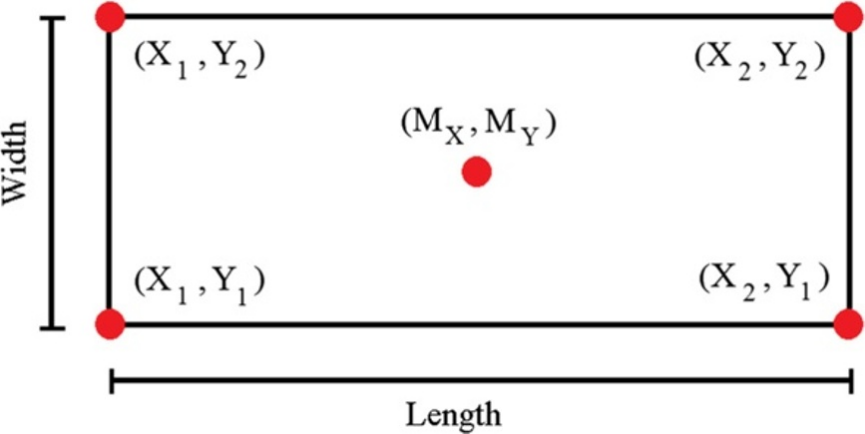
**Rectangle simulating the CFL.** Coordinates of the vertices of the simulated CFL, with length, width and center.

The image plane begins to be traced at the origin of the Cartesian system and ends at the coordinate X_F_ = 50 (the value chosen for graphical representation), with Y_F_ depending on the angular coefficient of the line (equation 1) that represents the image plane:
1

where: *α* = angle of the image plane.The dimensions of the rectangle (Figure 7) that represents the CFL are given by the following relationships:

The rectangle is rotated around the central point (M_X_, M_Y_) until it achieves the biometric position provided by the user. The matrix of rotation M is given by equation 9. 9

where: β = angle of maximum rotation of the CFL that permits complete visualization.

The “Processing” menu allows the user to choose the direction (clockwise or counter clockwise) of rotation of the CFL as a function of the image plane. The application calculates the new coordinates as a function of the ordinate axis (Y_rot_), using equation 10. 10

From Y_rot_, it is possible to represent a right triangle from the center of the rectangle to Y_rot_. Based on the right triangle, the hypotenuse and the opposite side can be calculated, and thus the angle of maximum rotation (clockwise and counter clockwise) of the representation of the CFL can be found. The expressions used in this phase are equations 11 and 12.
1112

**Figure 8:**
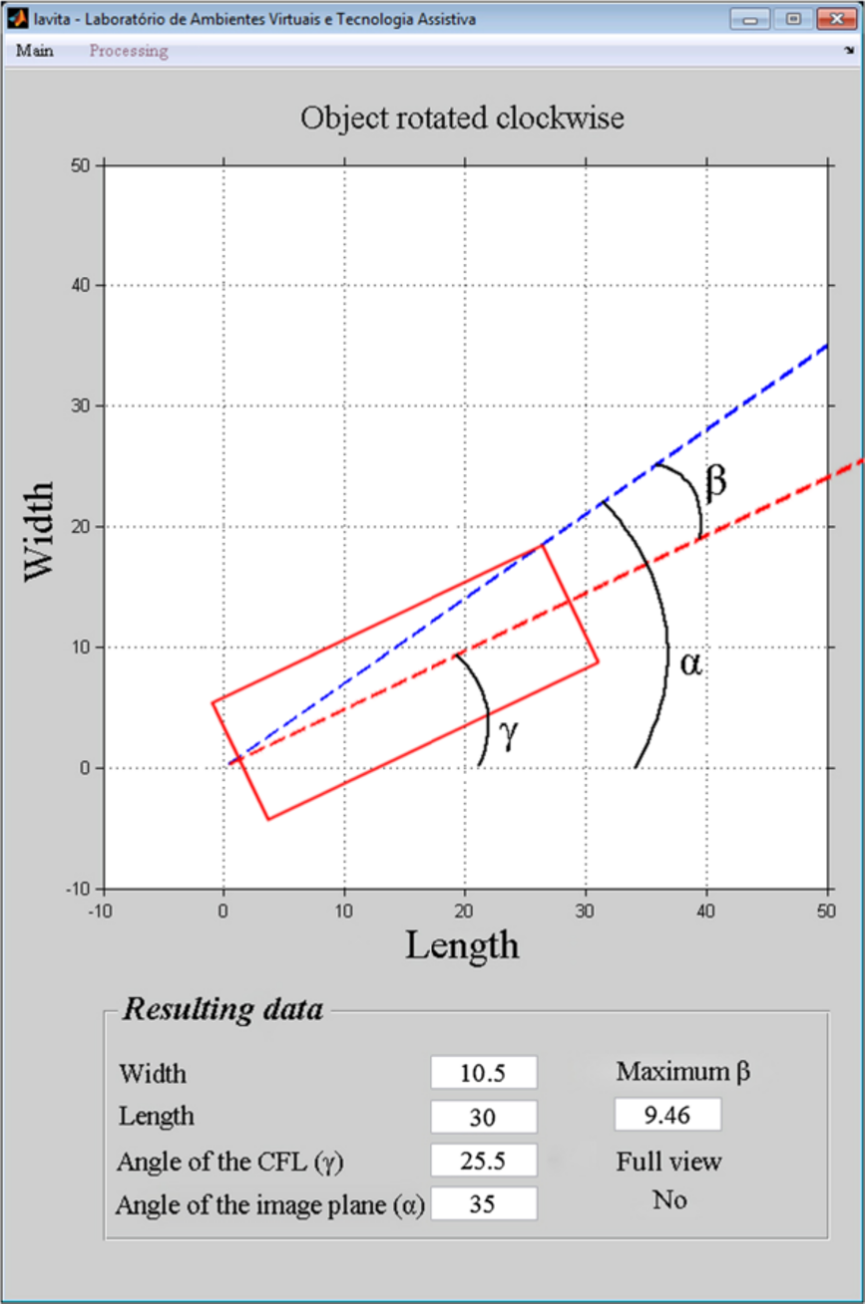
**Simulation of limit of the visualization complete of CFL.** Positioning of CFL to δ = 25,5°, image plane with α = 35° and the limit β angle for complete visualization of ligament.

As a result, the simulator shows the representation of the CFL (width and length), the angle of the image plane (α), the angle of the CFL (γ), the maximum angular variation as a function of the image plane (β), and whether complete visualization of this ligament occurred, as shown in Figure 8.

Another characteristic of the developed tool is the possibility of exporting the input and output data to a MS Excel® spreadsheet. This application also permits the printing of the graphical controls in conjunction with the data panels.

## Results

### Dimensions of the CFL

**Figure 9:**
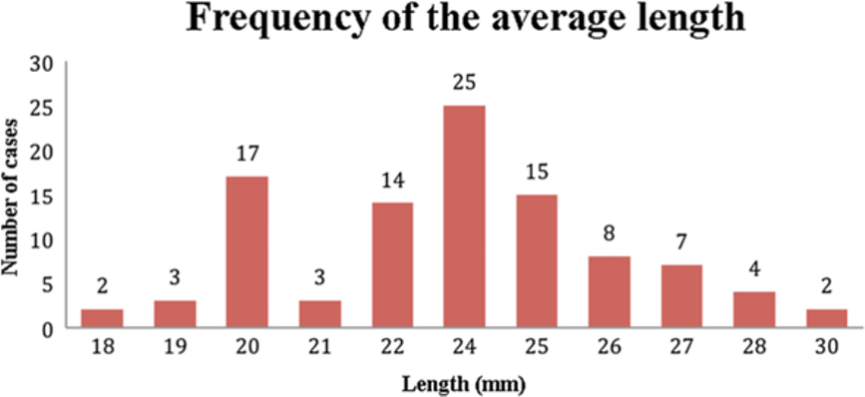
**Prevalence of the average length of the CFL.** CFL length distribution of 100 volunteers in the number of cases.

**Figure 10:**
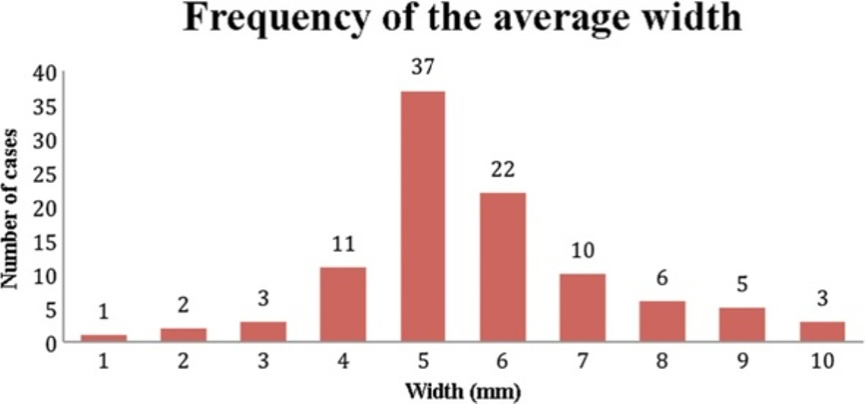
**Prevalence of the average width of the CFL.** CFL width distribution of 100 volunteers in the number of cases.

**Figure 11:**
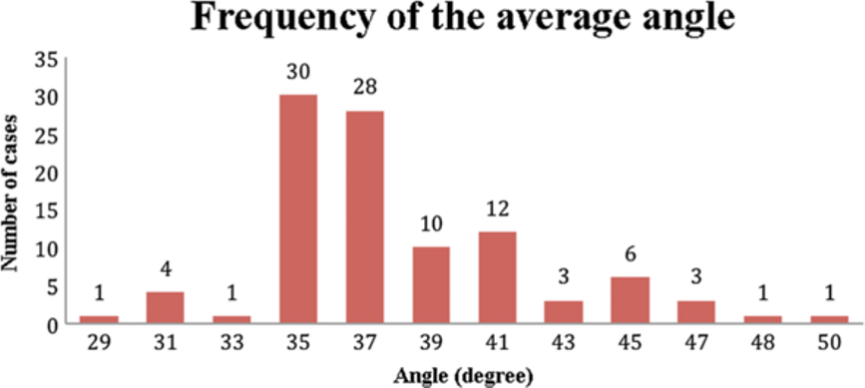
**Prevalence of the average angle of the CFL.** CFL angle distribution of 100 volunteers in the number of cases.

The averages of the measurements of the width (W), length (L), and angle of the CFL (γ) were calculated, thus the prevalence of these values (Figures 9, 10 and 11).

The average angle of the CFL from the sample varied from 29° to 50°, with the highest prevalence being at 35° and with 80% of the sample between 35° and 41°. The width of the CFL varied from 1 to 10 mm with the highest prevalence between 4 and 7 mm. The length varied from 18 to 30 mm, and the most frequent measurements were 20 mm (17 cases) and 24 mm (25 cases).

### Validation of the simulation

The reconstructed images and the simulations, both with the technique at 35°, where the complete CFL was not visualized, are in Table 1. The CFL was completely visualized in the other images and simulations. Based on these data, the accuracy of detection of the CFL was calculated, with the report from the doctors of the Lumen Diagnostics Centre considered to be the gold standard.

**Table 1:** **Accuracy in the reconstructed and simulated images**

Image	Average length (mm)	Average width (mm)	Average angle (degrees)	View of the evaluators	Visualization with simulator
14	30.00	5.00	46.00	0	0
25	23.10	5.00	48.30	0	0
26	23.00	1.50	41.03	1	0
30	22.90	5.07	50.07	0	0
83	30.00	2.00	29.00	0	0

Images where the entire CFL was not visualized (visualization 0): Averages for the length, width and angle with the oblique transversal technique at 35°.

**Figure 12:**
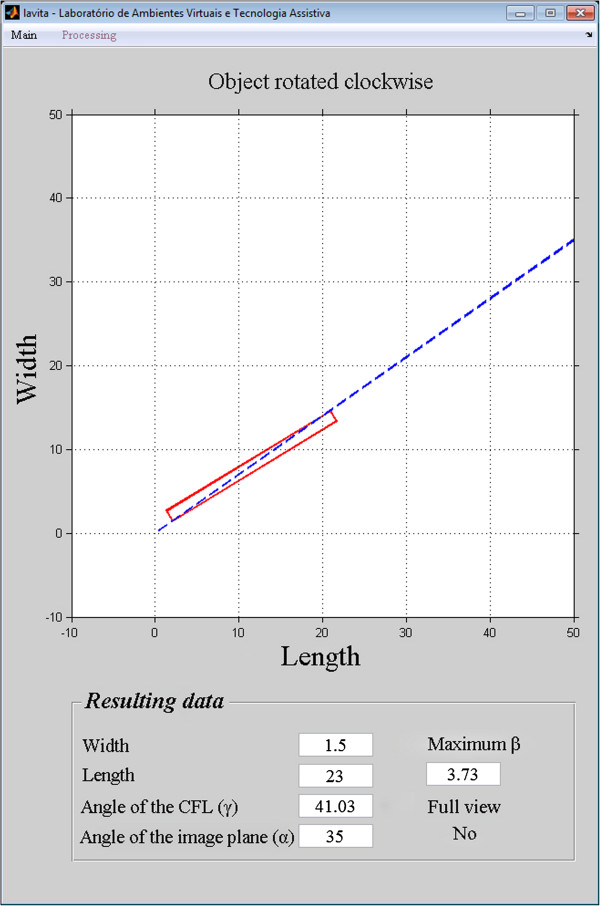
**Simulation of the image 26 with partial visualization of CFL.** Simulation of the image plane at 35° with CFL’s angle at 41.03°, resulting in the angular variation of 3.73°.

**Figure 13:**
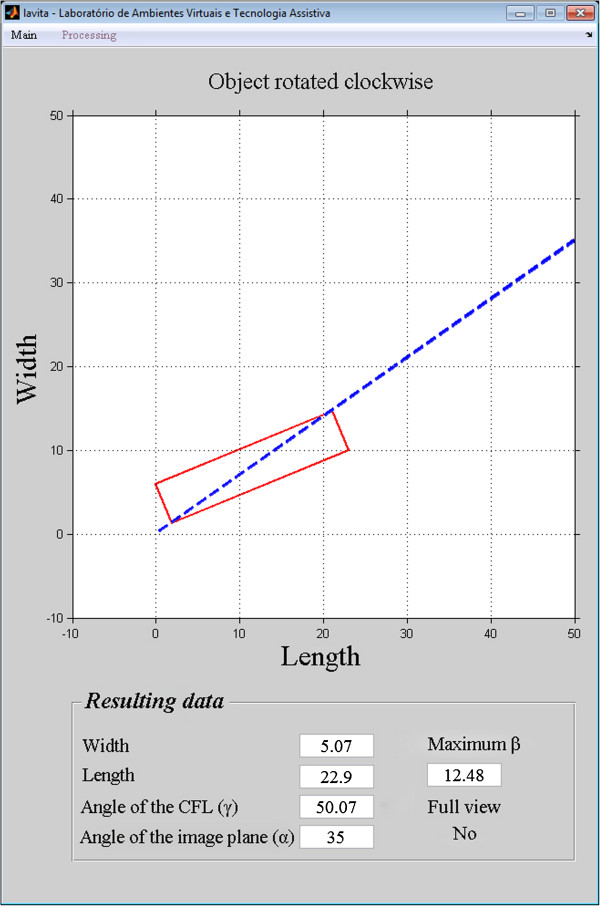
**Simulation of the image 30 with partial visualization of CFL.** Simulation of the image plane at 35° with CFL’s angle at 50.07°, resulting in the angular variation of 12.48°.

**Figure 14:**
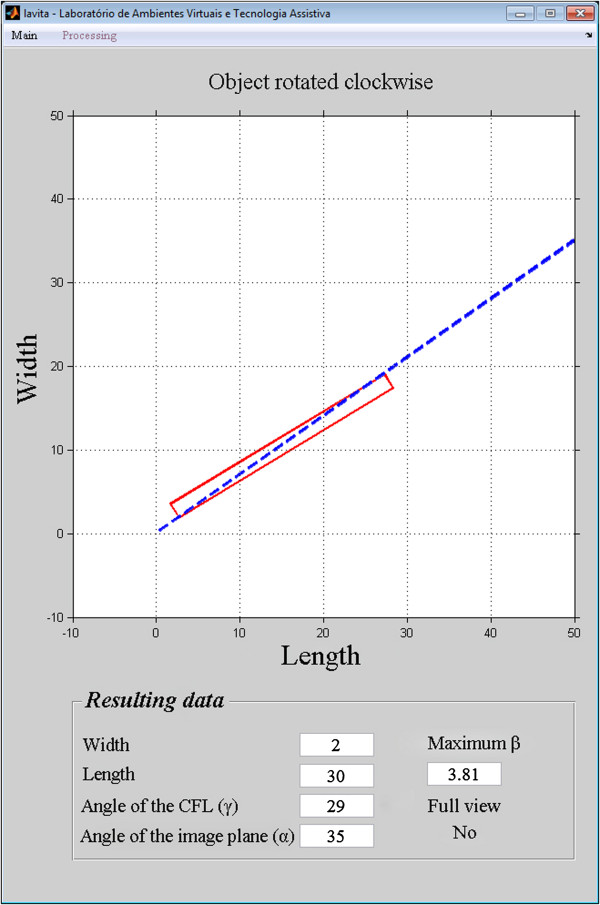
**Simulation of the image 83 with partial visualization of CFL.** Simulation of the image plane at 35° with CFL’s angle at 29°, resulting in the angular variation of 3.81°.

**Figure 15:**
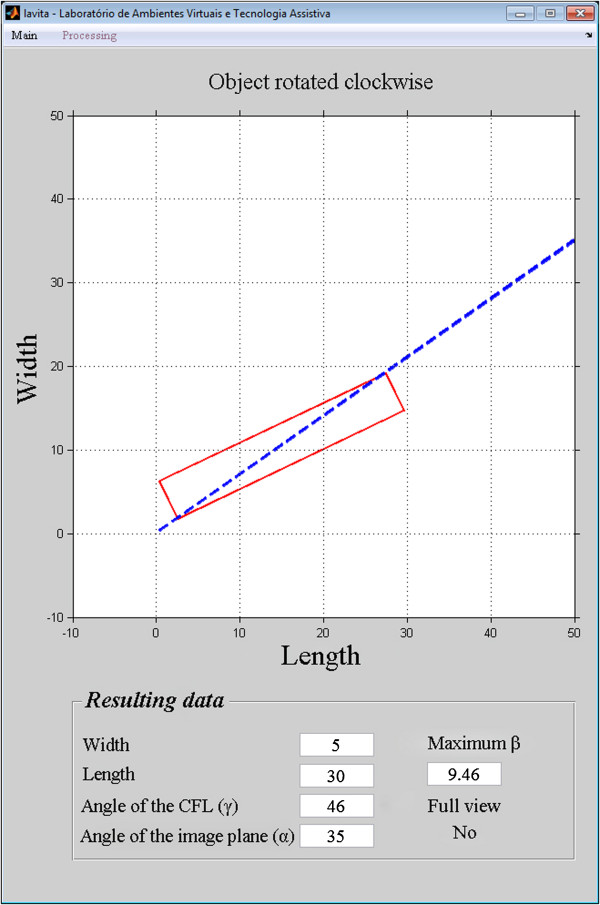
**Simulation of the image 14 with partial visualization of CFL.** Simulation of the image plane at 35° with CFL’s angle at 46°, resulting in the angular variation of 9.46°.

**Figure 16:**
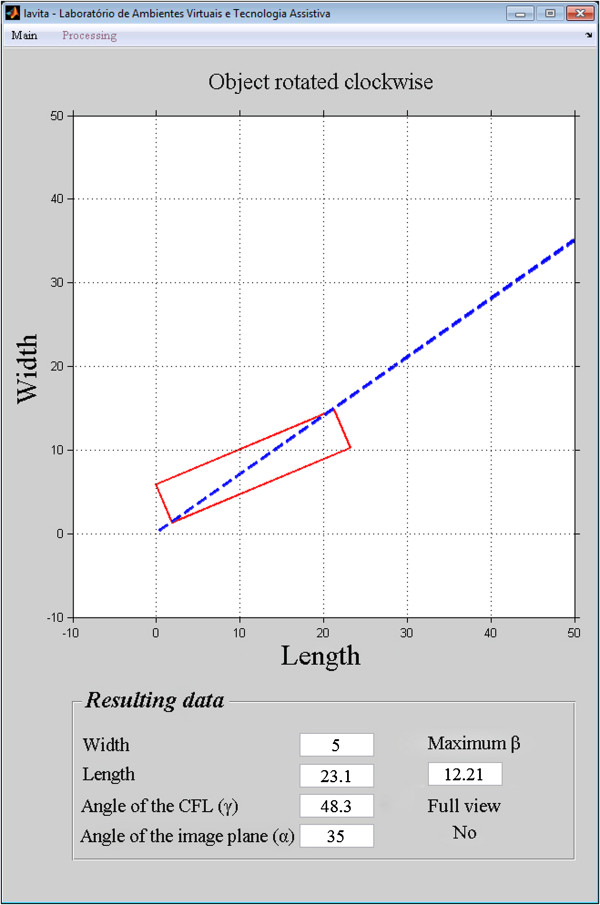
**Simulation of the image 25 with partial visualization of CFL.** Simulation of the image plane at 35° with CFL’s angle at 48.3°, resulting in the angular variation of 12.21°.

The accuracy of the images reconstructed at 35° was 96%, and the simulation accuracy at 35° was 95%. The 1% difference between the accuracy of the simulation and the accuracy of the reconstructed images shows that the model is valid for predicting the efficiency of the technique in the detection of the complete CFL.The position of the CFL that was not visualized in the simulation using the oblique transversal technique at 35°, shown in Figures 12, 13, 14, 15 and 16.

### Simulation of the maximum angular variation for all the oblique transversal techniques

Using the simulator, it was possible to obtain the maximum angular variation that allows the complete CFL visualization as a function of its dimensions for any angle of the oblique transversal image plane of the MR. Table 2 shows the value that must be added to or subtracted from the angle of the image plane to obtain the maximum and minimum of CFL for its complete visualization for all CFL length and width measurements. If the angle of the CFL is within these limits, it will be visualized.

**Table 2:** **Maximum angular variations generated by the simulator**

CFL width (mm)																	
CFL length (mm)	1.5	2	2.5	3	3.5	4	4.5	5	5.5	6	6.5	7	7.5	8	8.5	9	9.5
**21**	4.09	5.44	6.79	8.13	9.46	10.8	12.1	13.4	14.7	16	17.2	18.4	19.7	20.9	22	23.2	24.3
**22**	3.9	5.19	6.48	7.77	9.04	10.3	11.6	12.8	14	15.3	16.5	17.7	18.8	20	21.1	22.3	23.4
**23**	3.73	4.97	6.2	7.43	8.65	9.87	11.1	12.3	13.5	14.6	15.8	16.9	18.1	19.2	20.3	21.4	22.4
**24**	3.58	4.76	5.95	7.13	8.3	9.46	10.6	11.8	12.9	14	15.2	16.3	17.4	18.4	19.5	20.6	21.6
**25**	3.43	4.57	5.71	6.84	7.97	9.09	10.2	11.3	12.4	13.5	14.6	15.6	16.7	17.7	18.8	19.8	20.8
**26**	3.3	4.4	5.49	6.58	7.67	8.75	9.82	10.9	11.9	13	14	15.1	16.1	17.1	18.1	19.1	20.1
**27**	3.18	4.24	5.29	6.34	7.39	8.43	9.46	10.5	11.5	12.5	13.5	14.5	15.5	16.5	17.5	18.4	19.4
**28**	3.07	4.09	5.1	6.12	7.13	8.13	9.13	10.1	11.1	12.1	13.1	14	15	16	16.9	17.8	18.7
**29**	2.96	3.95	4.93	5.91	6.88	7.85	8.82	9.78	10.7	11.7	12.6	13.6	14.5	15.4	16.3	17.2	18.1
**30**	2.86	3.81	4.76	5.71	6.65	7.59	8.53	9.46	10.4	11.3	12.2	13.1	14	14.9	15.8	16.7	17.6
**31**	2.77	3.69	4.61	5.53	6.44	7.35	8.26	9.16	10.1	11	11.8	12.7	13.6	14.5	15.3	16.2	17
**32**	2.68	3.58	4.47	5.36	6.24	7.13	8	8.88	9.75	10.6	11.5	12.3	13.2	14	14.9	15.7	16.5
**33**	2.6	3.47	4.33	5.19	6.05	6.91	7.77	8.62	9.46	10.3	11.1	12	12.8	13.6	14.4	15.3	16.1
**34**	2.53	3.37	4.21	5.04	5.88	6.71	7.54	8.37	9.19	10	10.8	11.6	12.4	13.2	14	14.8	15.6
**35**	2.45	3.27	4.09	4.9	5.71	6.52	7.33	8.13	8.93	9.73	10.5	11.3	12.1	12.9	13.7	14.4	15.2

Value that must be added to or subtracted from the angle of the image plane for the widths of the CFL between 1.5 and 9.5 mm and lengths of 21 to 35 mm.

Figure 14 shows that 80% of the measurements of the angle of the CFL are between 35° and 41° in the studied population. Therefore, the MR techniques were simulated using the angles of 35, 37, 38, 39, 40, 41, and 45 degrees (Table 3), and the accuracy was calculated for each technique. The technique was simulated at 45° because this value is the most widely used in clinics. The degree of association between each simulated technique and the gold standard was calculated using the C contingency coefficient (Table 4). The accuracies for these simulated techniques showed that the best technique is to use the angle of the oblique transverse image plane between 38° and 41°. We selected 38° because it is closest to the angle of the CFL for the majority of the population.

**Table 3:** **Accuracy of the simulations**

Image	35°	37°	38°	39°	40°	41°	42°	45°
2	1	1	0	0	0	0	0	0
14	0	1	1	1	1	1	1	1
15	1	1	1	1	1	1	1	1
16	1	1	1	1	1	1	1	1
17	1	1	1	1	1	1	1	1
18	1	1	1	1	1	1	1	1
25	0	0	0	0	0	0	0	1
26	0	0	1	1	1	1	1	0
30	0	0	1	1	1	1	1	1
32	1	1	1	1	1	1	1	0
41	1	1	1	1	1	1	1	0
54	1	1	1	1	1	1	1	0
64	1	1	1	1	1	1	1	0
66	1	1	1	1	1	1	1	0
69	1	1	1	1	1	1	1	0
79	1	1	1	1	1	1	1	0
80	1	1	1	1	1	1	1	1
81	1	1	1	1	1	1	0	0
83	0	0	0	0	0	0	0	0
89	1	1	1	1	1	1	1	0
90	1	1	1	1	1	1	1	0
96	1	1	1	1	1	1	1	0
Accuracy	95%	96%	97%	97%	97%	97%	96%	87%

Detection of the full CFL in the simulations at 35°, 37°, 38°, 39°, 40°, 41°, 42° and 45°.

**Table 4:** **Agreement of the simulations with the gold standard**

	GS x 35°	GS x 37°	GS x 38°	GS x 39°	GS x 40°	GS x 41°	GS x 45°
Contingency coefficient (C)	0.1562	0.1400	0.1216	0.1216	0.1216	0.1216	0.2558
2 Columns							
Chi-squared	4.8750	3.9200	2.9550	2.9550	2.9550	2.9550	13.0200

Contingency coefficient (C) and chi-squared for 2 columns calculated between each simulated technique and the report from the doctors as the gold standard (GS).

Table 5 shows the complete visualization performed by the evaluators in the images reconstructed at 35°, 38°, and 45° as well as the simulated visualization for these techniques.

**Table 5:** **Comparison of the accuracy of CFL detection**

Image	Detection 35°		Detection 38°		Detection 45°	
	Real	Sim	Real	Sim	Real	Sim
2	1	1	1	0	1	0
14	0	0	1	1	1	1
16	1	1	1	1	0	1
17	1	1	1	1	0	1
19	1	1	1	1	0	1
20	1	1	1	1	0	1
25	0	0	1	0	1	1
26	1	0	1	1	1	0
28	1	1	1	1	0	1
30	1	0	1	1	1	1
31	1	1	0	1	1	1
32	1	1	1	1	1	0
36	1	1	1	1	0	1
41	1	1	1	1	1	0
45	1	1	1	1	0	1
54	1	1	1	1	1	0
64	1	1	1	1	1	0
65	1	1	1	1	1	1
66	1	1	1	1	1	0
67	1	1	1	1	1	1
68	1	1	1	1	1	1
69	1	1	1	1	1	0
78	1	1	1	1	0	1
79	1	1	1	1	1	0
81	1	1	1	1	1	0
83	0	0	0	0	0	0
89	1	1	1	1	1	0
90	1	1	1	1	1	0
96	1	1	1	1	1	0
Accuracy	97%	95%	98%	97%	91%	87%

Detection of the CFL in the reconstructed images, and detection in the simulations was performed at 35°, 38° and 45° angles, with (1) indicating that the ligament was visualized and (0) indicating that the ligament was not visualized.

The C contingency test was applied to relate 2 variables: the GS with the multiplanar reconstruction (MPR) and later the GS with the simulated data (Sim) for the 35°, 38°, and 45° angles. Both, considering a degree of freedom of 99 and p = 1, showing that the technique at 38° provides the closest result to the report from the doctors of the lumen clinic (Table 6).

**Table 6:** **Agreement between simulations, reconstructed images and gold standard, for two variables**

	GS x MPR 35°	GS x Sim 35°	GS x MPR 38°	GS x Sim 38°	GS x MPR 45°	GS x Sim 45°
Contingency coefficient (C)	0.1216	0.1562	0.0995	0.1216	0.2075	0.2558
2 Columns						
Chi-squared	2.9550	4.8750	1.9800	2.9550	8.5950	13.0200

Contingency coefficient (C) and chi-squared calculated between the GS and MPR as well as between the GS and simulations (Sim) obtained using the 35°, 38° and 45° angles, for two variables.

Additionally, the C contingency test was applied to relate 3 variables: the GS with the MPR and the Sim images for the 35°, 38°, and 45° angles, considering one degree of freedom = 198 and p = 1. Table 7 shows the degree of association among the variables.

**Table 7:** **Agreement between simulations, reconstructed images and gold standard, for three variables**

	GS x MPR x Sim 35°	GS x MPR x Sim 38°	GS x MPR x Sim.45°
Contingency coefficient (C)	0.1615	0.1285	0.2737
3 Columns			
Chi-squared	7.8168	4.9519	22.4315

Contingency coefficient (C) and chi-squared calculated between the GS and MPR as well as between the GS and simulations (Sim) obtained using the 35°, 38° and 45° angles, for three variables.

## Discussion

The average values obtained for the length, width, and angle of the CFL in this study are similar to the values obtained in the studies with cadaver samples performed by Golanó et al. [12], Taser et al. [15], Boonthathip et al. [10], and Yildiz and Yalcin [1]. However, the cadaveric conditions of the samples influenced the measurements, and the valgus or varus position of the stem considerably changes the angle formed by the ligament and the longitudinal axis of the fibula [12]. The MR method used in our study provided results as good as the previously validated techniques [1, 10, 12, 15]. But, the proposed approach can be easily applied allowing investigators to implement it extensively. In addition, our technique provided improved CFL dimensions when compared with the above cited approaches. However, subjective evaluation might contribute to distorted results. Other authors [16] showed that ankle morphological measurements are very sensitive to the positioning of the joint during imaging.

A study [17] evaluated the intra- and inter-observer variability in the determination of the locations of insertion and origin of the ligaments using MR images. The researchers showed that significant variation can occur in this location, with a considerable effect on the computational models. Therefore, to decrease the error in the insertion of the points of origin of the ligaments, in our study, the measurements were performed by a pair of professionals specializing in MR. The insertion was defined only when the two agreed. Despite this precaution, there can still be a certain amount of imprecision that may explain the 3% difference between the accuracy of the model and the standard shown in our study.

When a ligament is not completely visualized in the image, a lesion is diagnosed. However, the reliability of this discovery does not depend only on the lesion but also on the technical aspects of the magnetic resonance exam and the anatomical variations of the ligament. The use of an inappropriate image plane can provide images that lead to an incorrect diagnosis [5]. Other possible error source might be caused by the image acquisition due to the multiplanar reconstruction (MPR) technique. This technique was adopted to allow multiple image planes without the need of the volunteer undergo the prolonged procedure. However, to avoid in presenting a partial perspective in relation to other alternatives, we as a start did the first image acquisition using the oblique technique at 35° and the authors observed that both CFL complete visualization and isotropies images volumetric reconstruction at 35° presented equal accuracy.

Various researchers have sought to improve the complete detection of the CFL. Initially, the studies investigated the best image plane and foot position to avoid false positive. Beltran et al. [6] used the coronal plane and the foot of the volunteers in plantar flexion and obtained complete visualization in 81% of normal cases. Park et al. [2], using axial and sagittal MR, showed 90% precision in visualization. Pastore et al. [7] studied MR in the axial and coronal planes and obtained only partial visualization of the CFL. Joshy et al. [8] used MR with an axial, coronal, and sagittal sequence that exhibited 87.5% precision.

A study performed by Boonthathip et al. [10] showed that the results improve when the image plane is aligned with the angle of the CFL and proved that this angle can be different for each person. Therefore, the angle of the image plane should be adjusted for each person. However, the width and length of the CFL can vary up to 50% among individuals, and this factor had not been considered. The model developed in this study permitted the development of a technique that serves the majority of patients without causing false positive in the detection of lesion of the CFL.

This study did not address the cases of false negatives in patients with suspicions of lesion of the CFL or cases of CFL partially torn.

## Conclusion

This MR technique is proposed to improve the diagnosis for the majority of patients with suspicions of CFL ankle lesions, minimizing the possibility of false positives. This technique determine the most appropriate image plane. The choice of MR as a tool to measure CFL is due to the lack of protocols for CFL ankle lesions. In addition, this approach provided better sensitivity to investigate correlates impairment such as degenerative, traumatic, and ligament diseases of the ankle.

In future studies we will analyze the oblique transversal technique of the ankle at the angle of 38° for patients with CFL lesions.
